# Feasibility of using a handheld ultrasound device to detect and characterize shunt and deep vein thrombosis in patients with COVID-19: an observational study

**DOI:** 10.1186/s13089-020-00197-0

**Published:** 2020-11-30

**Authors:** Rajkumar Rajendram, Arif Hussain, Naveed Mahmood, Mubashar Kharal

**Affiliations:** 1 Department of Medicine, King Abdulaziz Medical City, King Abdullah International Medical Research Center, Ministry of National Guard - Health Affairs, Riyadh, Saudi Arabia; 2 College of Medicine, King Saud Bin Abdulaziz University for Health Sciences, Riyadh, Saudi Arabia; 3 Department of Cardiac Sciences, King Abdulaziz Medical City, King Abdullah International Medical Research Center, Ministry of National Guard - Health Affairs, Riyadh, Saudi Arabia

**Keywords:** COVID-19, SARS-CoV-2, Point-of-care ultrasound, Lung ultrasound, Echocardiography, Deep vein ultrasound, Handheld ultrasound device, Shunt, Patent foramen ovale

## Abstract

**Background:**

Coronavirus disease 2019 (COVID-19) causes an atypical acute respiratory distress syndrome associated with thromboembolism and high shunt fraction. Shunt may be intrapulmonary, or extrapulmonary. Handheld devices are increasingly being used for point-of-care ultrasound, but their use to characterize shunt has not been reported.

**Objectives:**

Determine the feasibility of using handheld ultrasound to detect and characterize anatomical substrates of hypoxia and deep vein thrombosis (DVT) in patients with COVID-19 suspected to have severe shunt.

**Methods:**

A handheld ultrasound device (iQ, Butterfly, USA) was used to perform lung ultrasound, vascular assessment for DVT, and limited transthoracic echocardiography (TTE) with color Doppler and saline microbubble contrast in patients with COVID-19 suspected to have severe shunt. Images were reassessed by an independent reviewer.

**Results:**

After screening 40 patients, six patients who fulfilled the inclusion criteria were identified. Two were excluded because palliation had been initiated. So, four patients were studied. Interpretable images were obtained in all cases. Interobserver agreement was good. All patients had abnormal lung ultrasound (lung ultrasound score range 17–22). Identified lung pathology included interstitial syndrome with light beams and small peripheral consolidation (4), lobar consolidation (1), and pleural effusion (1). Abnormal echocardiographic findings included interatrial shunt (2), intrapulmonary shunt (1), and dilated right ventricle with tricuspid valve regurgitation (1). Significant DVT was not detected.

**Conclusion:**

Use of handheld ultrasound to perform combined lung ultrasound, DVT ultrasound, and limited TTE with color Doppler and saline microbubble contrast is feasible, and may be able to characterize shunt in critically hypoxic patients. Serial studies could be used to monitor changes in shunt. Further studies are required to determine whether this can guide treatment to improve the outcomes of patients with refractory hypoxia.

## Background

The acute respiratory distress syndrome (ARDS) caused by Coronavirus Disease 2019 (COVID-19) is atypical [1–3]. Indeed, two distinct patterns of COVID-19 pneumonia (type L, low elastance and type H, high elastance) have been described [2].

The pathogenesis of COVID-19 involves pulmonary microvascular thrombosis, and venous thromboembolism is very common [3, 4]. While this should increase dead space, one series of critically ill patients with COVID-19 had a high mean right-to-left (RTL) shunt fraction (0.50 ± 0.11) despite relatively normal lung compliance (50.2 ± 14.3 ml/cmH_2_O) [1]. The pathogenesis of the dead space and shunt in COVID-19 is likely to be multifactorial [5]. Shunt may be intrapulmonary (IPS; e.g., lobar collapse or consolidation, ARDS, pulmonary arteriovenous malformations, hepatopulmonary syndrome), or extrapulmonary (e.g., intracardiac shunts) [5, 6]. Indeed, one study using lung ultrasound enhanced with sulfur hexafluoride microbubble contrast (SonoVue), demonstrated the presence of partial, inhomogeneous, regions of intrapulmonary hyperperfusion in patients with COVID-19 pneumonia [3].

The cardiac dysfunction associated with COVID-19 may vary with the respiratory phenotype [7]. For example, the high airway pressures required to ventilate patients with phenotype H are likely to have a significant effect on right heart function [7]. In contrast, as patients with phenotype L have relatively preserved lung compliance, the airway pressures required to achieve the set tidal volumes during the mechanical ventilation should be low [7]. So, less right heart impairment is expected in patients with phenotype L COVID-19 pneumonia.

Precise measurement of dead space, IPS, extrapulmonary shunt, and right heart function requires left, and right heart catheterization. However, this is too invasive to be used for screening. An alternative strategy to assess left and right heart function and screen for RTL shunt could include transthoracic echocardiography (TTE).

In spontaneously breathing, breathless patients with phenotype L, TTE may demonstrate the consequences of increased respiratory effort on ventricular interdependence. Extremely negative intrathoracic pressures may reduce stroke volume if diastolic ventricular septal shift results in right ventricular (RV) overload and left ventricular (LV) hypodiastole. In patients with phenotype H receiving positive pressure mechanical ventilation, TTE may detect cardiac complications of ventilatory support. These include RV dysfunction (i.e., RV dilation, tricuspid regurgitation, reduced RV systolic function), and secondary LV dysfunction (i.e., acute cor pulmonale) [7, 8]. Moreover, it is important to recognize that ventilator-induced heart failure can occur in patients with previously normal cardiac function [9].

Furthermore, TTE with color Doppler, and saline microbubble contrast [10, 11], a minimally invasive test, can detect and quantify shunt, and distinguish IPS from intracardiac shunt [10, 11]. Lung ultrasound can detect pulmonary pathology that may increase IPS [12]. Vascular ultrasound can detect deep venous thrombosis (DVT), a precursor for pulmonary embolism (PE). While PE is not universally present in patients with DVT, in the presence of DVT, anticoagulation is indicated to prevent PE. The detection of DVT is therefore important in patients with severe hypoxia. Thus, combined lung ultrasound, DVT ultrasound, and limited TTE with color Doppler and saline microbubble contrast could detect and characterize the substrates for severe hypoxia.

A previous study demonstrating the feasibility of performing limited TTE, and lung ultrasound, in critically ill patients, used a cart-based system [13]. Handheld ultrasound devices have several advantages over cart-based systems [14–16], particularly in the context of COVID-19 [16]. Yet, despite their widespread use, it is not known whether the image quality obtained from handheld devices is adequate to determine the etiology of shunt.

### Aim

Determine the feasibility of using a handheld ultrasound device to detect and characterize the substrates for hypoxia and DVT in patients with COVID-19 pneumonia suspected to have severe shunt.

## Methods

### Setting

This prospective, observational study was approved by the institutional review board of the King Abdullah International Medical Research Center, Riyadh, Saudi Arabia (Reference RC20/296/R). The need for formal patient consent was waived as written consent for critical care included use of ultrasound and TTE.

The study took place in sites designated as COVID-19 high dependency (HDU), and intensive care units (ICU) in a 1600-bed, tertiary-care hospital, Riyadh, Saudi Arabia.

### Protocol

Inclusion criteria were participants aged over 18 years, admitted to HDU or ICU, with COVID-19, in whom significant shunt was suspected. Significant shunt was suspected if any of the following criteria were present [6]:

1. Known anatomical substrate for shunt (e.g., patent foramen ovale; PFO).
2. Acute stroke or systemic thromboembolism.
3. Platypnea-orthodeoxia.
4. PaO_2_/FiO_2_ < 200 (estimated shunt fraction > 0.2).
5. The lung injury present on imaging did not fully account for hypoxia.
6. Supplemental oxygen did not significantly improve hypoxia.
7. Significant acute drop in the PaO_2_/FiO_2_ ratio.

Patients were excluded if palliation had been initiated, or was imminent.

Sample size calculation: with a prevalence of 20–30% in the general population, the most common cause of shunt is PFO [17, 18]. To identify five patients with an anatomical substrate for shunt it was estimated that at least 20 patients would have to be screened using the criteria above. To allow for exclusion of up to 50% of patients, it was decided that at least 40 patients should be screened for imaging with ultrasound. To identify suitable participants, patients’ electronic medical records were screened by A.H. and R.R. between 01/07/20 and 08/07/20 whenever both were available (convenience sampling).

Lung ultrasound, DVT ultrasound, and limited TTE with color Doppler and bubble contrast were performed by A.H. and R.R. Both are intensivists, accredited in full or limited echocardiography and point-of-care ultrasonography, with more than 15 years of imaging experience and institutional privileges. The imaging protocols used in our study were similar to previously described protocols [13, 19–23].

### Devices used for imaging

A handheld, application-based, ultrasound device (iQ, Butterfly, USA) with a compatible smartphone (iPhone 10S, Apple, USA) was used for imaging. A cart-based ultrasound machine (Vivid I, General Electric Healthcare, USA) was immediately available for comparative imaging in case diagnostic quality images could not be obtained with the handheld device.

### Lung ultrasound

Lung ultrasound was performed with the patient semi-recumbent (15° head elevation), or prone. The pleura and lungs were imaged at points in six zones for each lung: anterior upper, anterior lower, lateral upper, lateral lower, posterior upper, and posterior lower [13, 24–26].

A normal lung pattern was defined by the presence of smooth, thin pleural line with normal lung sliding, A-lines (pleural reverberation artifacts), and less than three B-lines (hyperechoic, vertical artifacts arising from the pleural line, reaching the bottom of the screen, and possibly fading or obliterating A-lines, moving with lung sliding) [13, 21].

The presence of 3, or more, B-lines, in a single intercostal space, in a non-dependent region was considered abnormal (B-Pattern). This could indicate pulmonary edema, pneumonitis, atelectasis, or contusion according to clinical context, and distribution of LUS findings in other regions. Interstitial syndrome was defined as B-pattern involving 2, or more, regions of both lungs [13, 21].

Poorly echoic, or isoechoic tissue-like (“hepatized”) areas (either peripheral and wedge-shaped, or more extended, deeper with sharp peripheral margins) identified consolidations (C-Pattern) [13, 21]. Alveolar consolidation was differentiated from atelectasis by the presence of dynamic air bronchograms. Fluid bronchograms, static air bronchograms or absent bronchograms characterized atelectasis. Color Doppler was not used to assess C-Patterned lung because the limited capability of the color Doppler on the handheld device is insufficient to detect very low velocity blood flow such as may be present in consolidated lung.

Pneumothorax was defined in the absence of lung sliding, B-lines, lung pulse, and C-Pattern and, the presence of lung point in more dorsal areas [13, 21]. Pleural effusion was defined as an anechoic space between the parietal and visceral pleura [21].

‘Small peripheral consolidation’ was diagnosed in the presence of a small, peripheral area of consolidated lung tissue surrounded by multiple B-lines, and an irregular or fragmented pleural line [13, 21]. Bilateral areas of B-patterned lung (i.e., interstitial syndrome), with multiple, patchy, small peripheral consolidations, alternating with large, well demarcated, “spared” areas have recently been described in patients with COVID-19 pneumonia [22, 27].

### Vascular ultrasound assessment for deep vein thrombosis

Screening for DVT using 2-D imaging, color Doppler, and compression ultrasonography, was based on two-point protocols for assessment of lower limb veins (common femoral, and popliteal) [19]. However, as the risk of thrombosis in COVID-19 is extremely high [4], upper limb veins (jugular, and subclavian) were also assessed.

### Limited TTE with color Doppler

The limited TTE examination generally followed the methods described for Focused Cardiac Ultrasound (FoCUS) [23]. This is a simplified, problem-oriented, mostly qualitative two-dimensional (2-D) TTE examination performed by clinicians. The study uses five echocardiographic windows (parasternal long-axis, parasternal short-axis, apical four-chamber, subcostal four-chamber, and inferior vena caval view) [23]. We also used color Doppler, to assess gross valve abnormalities, and screen for intracardiac shunt. The presence, or absence, of impaired left ventricular (LV) function, LV dilatation, right ventricular (RV) dilatation, impaired RV function, valve stenosis or regurgitation, intracardiac shunt, pericardial effusion, or hypovolemia were documented.

### Assessment of shunt using intravenous saline microbubble contrast

Saline microbubble contrast echocardiography was performed to detect interatrial shunt, and IPS. Saline microbubble contrast (10 ml) was prepared by agitation of normal saline (9 ml) with air (1 ml) between two Luer lock syringes connected via a 3-way stopcock [6, 11]. After obtaining a clear 4-chamber view (subcostal or apical), the saline microbubble contrast was injected intravenously (i.v.). If present, a central venous catheter was used in preference to peripheral venous catheters.

The diagnosis of shunt was made if the contrast was visualized in the left heart after opacification of right heart. Intracardiac shunt was diagnosed if contrast appeared in the left heart within 3 cardiac cycles of entering the right heart [6, 11]. If contrast required 4–6 cardiac cycles to reach the left heart, IPS was suspected [6, 11]. Cardiac cycles were counted by contractions as electrocardiography was not available on the handheld device.

Shunt was graded by the maximum number of bubbles seen in a single chamber of the left heart on a single still frame [10]. Grade 1: < 5 bubbles; grade 2: 5 to 25 bubbles; grade 3: > 25 bubbles; and grade 4: opacification of chamber [10].

Various maneuvers increase the sensitivity of screening tests for intracardiac shunt [28]. If no shunt was detected after the first injection, it was repeated on release of a Valsalva maneuver (spontaneously breathing patients), or on release of an inspiratory hold (mechanically ventilated patients).

### Safety of imaging procedures with saline microbubble contrast in critically ill patients

To detect complications, patients were monitored continuously with at least pulse oximetry, non-invasive blood pressure, and three-lead electrocardiography, during and after imaging procedures. Patients were reviewed 24 h post-imaging for evidence of complications that may have been related to imaging, or injection of saline microbubble contrast.

### Reporting and re-assessment of images

Lung ultrasound, DVT ultrasound, and limited TTE findings were agreed by the imaging intensivists while scanning. Ultrasound findings were then documented on structured report forms. If any scans could not be performed, this, and the reason for not being able to complete the ultrasound examination, was also recorded. A lung ultrasound score [25, 26] was used to quantify the severity of abnormalities detected on lung ultrasound. Each of the 12 zones imaged was graded on a scale from 0–3 based on the presence or absence of various specific sonographic findings Thus, the highest possible score was 36.

An independent observer (N.M.), accredited in full TTE, with over 10 years of imaging experience, blinded to all patient data, reviewed the TTE images and reports offline, for quality, interpretability, and accuracy of diagnosis within 6 h of imaging.

Digital images of at least 5 cardiac cycles for each standard TTE view and at least 10 cardiac cycles for each saline microbubble contrast study were assessed offline after download to a desktop computer from the Butterfly Cloud (Butterfly, USA). If the TTE diagnosis differed substantially between the initial report, and the review, discussion between A.H., R.R., and N.M. was used to resolve the discrepancies.

## Results

Forty patients were screened to identify six who fulfilled the inclusion criteria. Two were excluded as palliative management had been initiated. Four patients (mean age 64.25 years; 3 women; 2 mechanically ventilated via endotracheal tubes, 2 receiving high-flow nasal oxygen) were recruited because their PaO_2_/FiO_2_ ratios were under 200, and there was clinical suspicion that the lung injury seen on chest X-ray did not fully account for this hypoxia. All patients improved with supplemental oxygen, and prone positioning. None had platypnea-orthodeoxia, or acute systemic embolism, and none were known to have an anatomical substrate for shunt prior to the study.

Complete examinations with lung ultrasound, DVT ultrasound, and limited TTE with color Doppler and saline microbubble contrast were performed on all participants. The ultrasound findings are correlated with patients’ respiratory support and outcomes in Table 1. While two patients subsequently died, there were no immediate or delayed respiratory or hemodynamic complications related to imaging, or injection of saline microbubble contrast.

**Table 1:** Correlation of respiratory support with findings on imaging and outcomes

Subject	1	2	3	4
Respiratory support	High-flow nasal canula		Mechanical ventilation VC (AC) via endotracheal tube	
FiO_2_	0.6	0.7	1.0	1.0
Flow (l/min)	60	60	–	–
PEEP (cmH_2_O)	–	–	10	10
Tidal volume set/achieved (ml)	–	–	370/374	330/265
Peak airway pressure (cmH_2_O)	–	–	34	32
Dynamic compliance (ml/cmH2O)	–	–	15.6	12
SpO_2_	88%	89%	98%	97%
PaO_2_/FiO_2_	111	61	64	82
Lung ultrasound score	22	20	17	20
Lung ultrasound findings	Interstitial syndrome with pleural line thickening and irregularities, in all 12 zones, with light beams, and bilateral patchy areas with small peripheral consolidations			
			Left lobar consolidation, and moderate left pleural effusion	Bibasal atelectasis
DVT ultrasound	Negative	Negative	Negative	Negative
Echocardiography	Normal	Normal	Dilated RV with impaired function	Normal
Saline microbubble contrast study	Negative	Grade 2 interatrial shunt	Grade 4 interatrial shunt	Grade 3 intrapulmonary shunt
Day of imaging (after admission)	Day 9	Day 8	Day 14	Day 14
COVID-19 phenotype			H (high elastance)	H (high elastance)
Outcome	Discharged home Day 17	Intubated Day 10, discharged home Day 39	Death Day 17	Death Day 16

Shunt grading: grade 1: < 5 bubbles; grade 2: 5 to 25 bubbles; grade 3: > 25 bubbles; and grade 4: opacification of chamber [10]. *DVT* deep vein thrombosis, *PEEP* positive end expiratory pressure, *RV* right ventricle, *VC (AC)* volume control (assist control). Phenotypes of COVID-19 have been described: L (low elastance) and H (high elastance)

Image quality was considered to be interpretable by all the study sonographers. Use of the Vivid I ultrasound machine was not required. There were no disagreements between the study sonographers in any of the ultrasound findings. As a result, all investigators considered that the objectives of the present study had been fulfilled, and further investigation was deemed unnecessary.

### Lung pathology

**Fig. 1:**
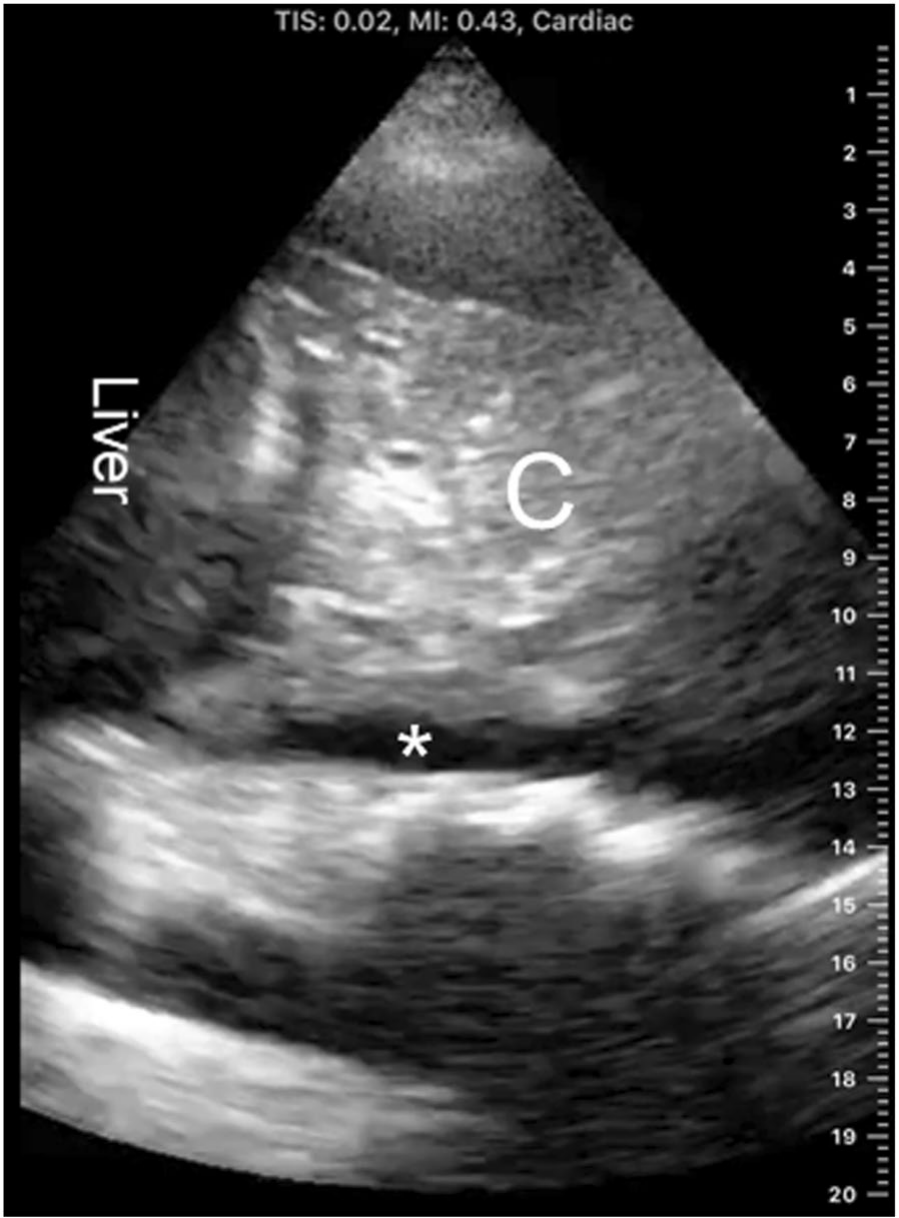
Consolidated lung in a mechanically ventilated patient with intrapulmonary shunt. Consolidated lung in a mechanically ventilated patient with intrapulmonary shunt. Lung ultrasound demonstrating a large area of consolidated lung (C) surrounded by a moderate pleural effusion (*) in zone 6 of the right lung

Interstitial syndrome with ‘light beams’ and small peripheral consolidations was diagnosed in all patients. One patient had lobar consolidation with a moderate pleural effusion (Fig. 1). These abnormalities may represent the anatomical substrate for increased dead space and IPS.

### Ultrasound assessment for deep vein thrombosis

Adequate views of internal jugular, subclavian, femoral and popliteal veins were obtained in all patients. However, 2-D imaging, color Doppler, and compression ultrasonography did not demonstrate any significant thrombus.

### Limited TTE with color Doppler

**Fig. 2:**
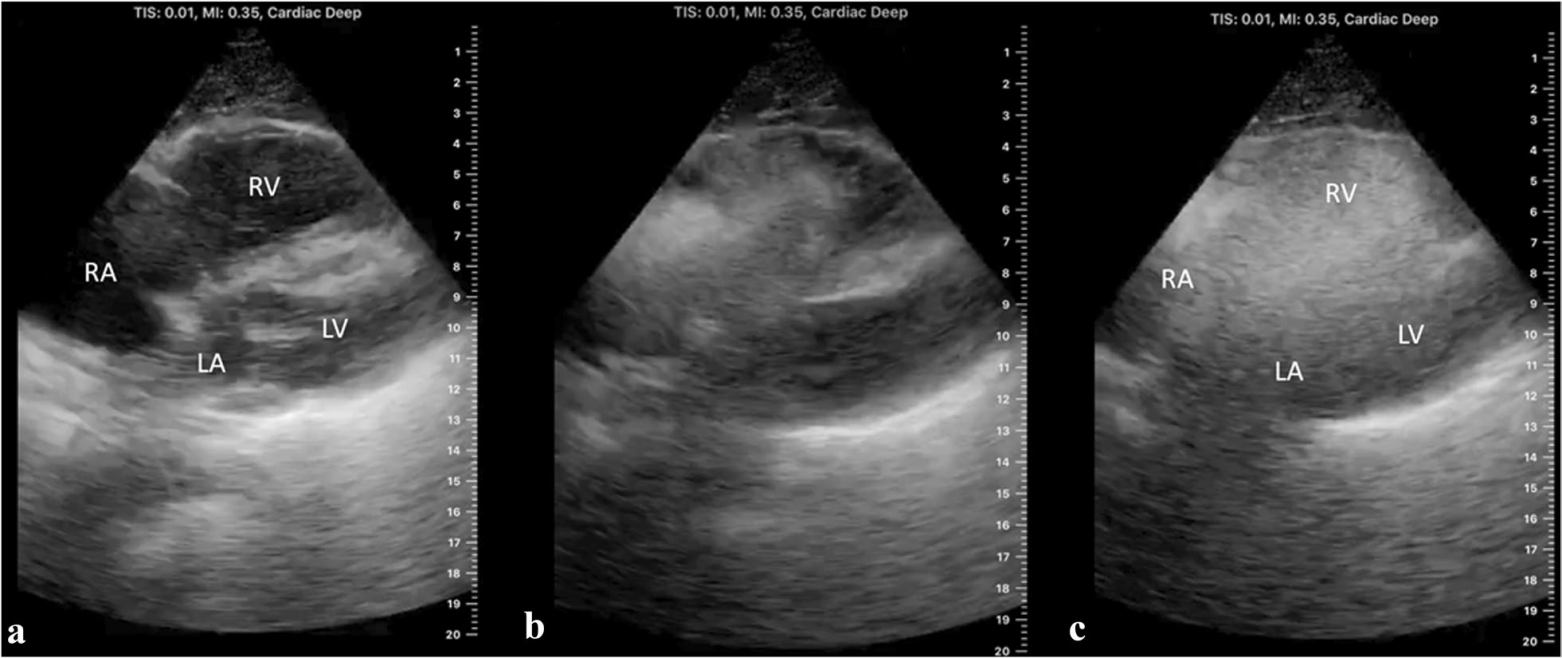
Interatrial shunt in a mechanically ventilated patient with acute cor pulmonale. Interatrial shunt in a mechanically ventilated patient with acute cor pulmonale due to COVID-19. **a** Labeled 2-D subcostal 4-chamber transthoracic echocardiographic (TTE) view of the heart demonstrating dilatation of the right atrium (RA) and right ventricle (RV). LA, left atrium; LV, left ventricle. **b** 2-D subcostal 4-chamber TTE view of the heart after i.v. injection of saline microbubble contrast showing the arrival of the contrast in the RA and RV. **c** Labeled 2-D subcostal 4-chamber TTE view of the heart after i.v. injection of saline microbubble contrast demonstrating grade 4 shunt (opacification of left atrium). Bubbles appeared in the LA within 3 cardiac cycles of opacification of the RA demonstrating the presence of an interatrial shunt

Hemodynamic state abnormalities were identified with limited TTE in only one participant. This mechanically ventilated patient had a dilated right ventricle (Fig. 2) with poor function due to acute cor pulmonale.

### Defining the etiology of shunt using color Doppler and saline microbubble contrast

Adequate subcostal views were obtained in all patients. Adequate apical 4-chamber views were obtained in two patients. Application of color Doppler did not demonstrate intracardiac shunt in any of the patients. However, it is important to note that the capability of the color Doppler on the handheld device is limited.

The subcostal views were used for all i.v. saline microbubble contrast studies. In two patients, microbubbles were detected in the left heart within 3 cardiac cycles of opacification of the right heart. Of these patients, a mechanically ventilated patient with acute cor pulmonale had grade 4 interatrial shunt (Fig. 2c; Additional file 1: Video S1), and a spontaneously breathing patient on high-flow nasal canula had grade 2 interatrial shunt, but TTE was otherwise unremarkable. It is important to note that when grade 3 or 4 interatrial shunt is detected with contrast-enhanced echocardiography, co-existing IPS cannot be excluded.

**Fig. 3:**
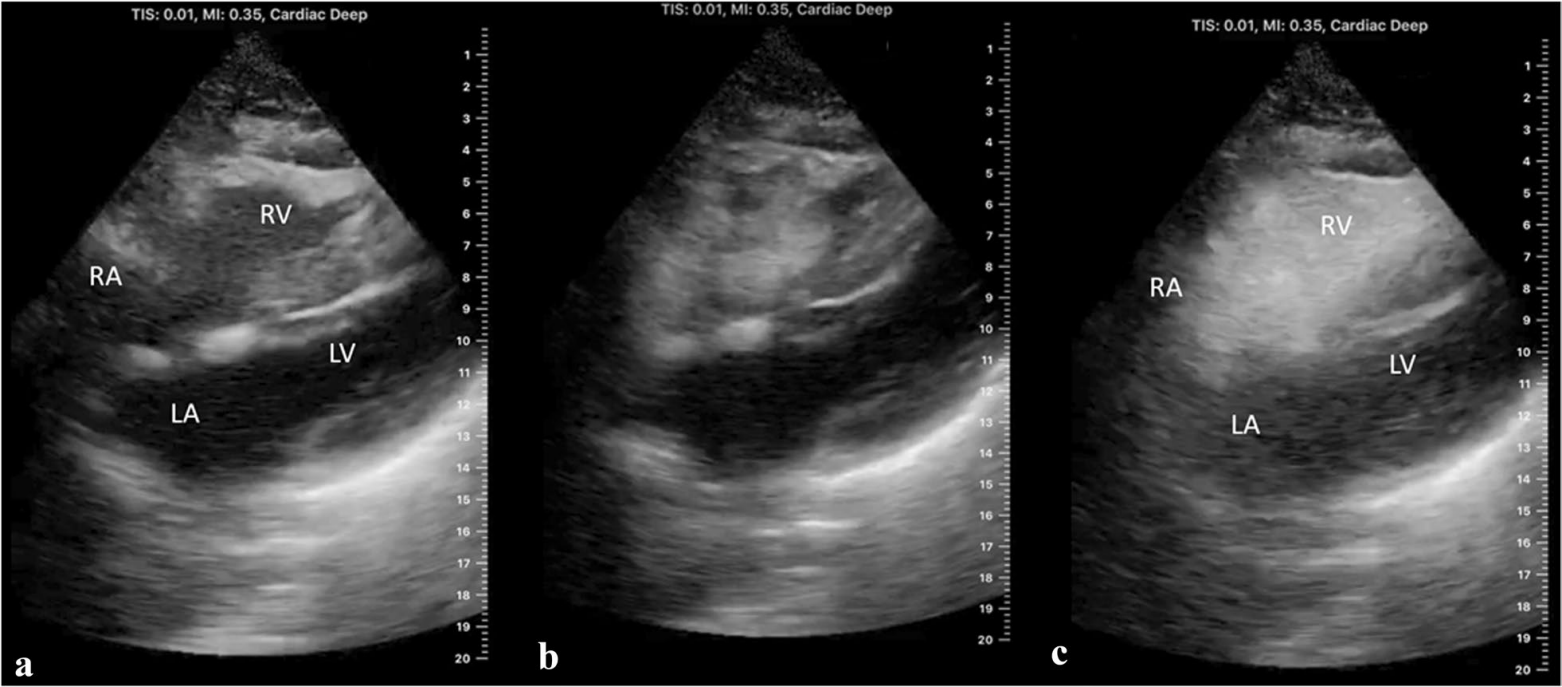
Intrapulmonary shunt in a mechanically ventilated patient with consolidated lung. Intrapulmonary shunt in a mechanically ventilated patient with consolidated lung. a Labeled 2-D subcostal 4-chamber transthoracic echocardiographic (TTE) view of the heart. *RA* right atrium, *RV* right ventricle, *LA* left atrium, *LV* left ventricle. b 2-D subcostal 4-chamber echocardiographic view of the heart after i.v. injection of saline microbubble contrast showing the arrival of the contrast in the RA and RV. c Labeled 2-D subcostal 4-chamber echocardiographic view of the heart after i.v. injection of saline microbubble contrast demonstrating grade 3 shunt (> 25 bubbles). Bubbles appeared in the left ventricle 5 cardiac cycles after opacification of the right ventricle suggesting the presence of an intrapulmonary shunt

In the other mechanically ventilated patient, > 25 microbubbles were detected (grade 3 shunt; Fig. 3; Additional file 2: Video S2) in the left heart 5 cardiac cycles after opacification of the right heart. This suggested IPS which may have been related to the presence of lobar consolidation (Fig. 1) in COVID-19 pneumonia [3]. A single injection of contrast was sufficient to demonstrate the shunt in these patients. These three patients did not require any maneuvers to increase the sensitivity of TTE for investigation of their RTL shunts.

After i.v. injection of saline microbubble contrast, no microbubbles were detected in the left heart of the other patient breathing spontaneously on high-flow nasal cannula. This patient received a second i.v. injection of saline microbubble contrast on release of a Valsalva maneuver. As this was also negative, IPS could still contribute to the severe hypoxia in this patient.

The pathogenesis of COVID-19 is thought to involve pulmonary endothelialitis, microvascular thrombosis, and dysregulation of hypoxic pulmonary vasoconstriction [3, 4]. This may cause very small IPS which cannot be excluded by negative saline microbubble contrast-enhanced echocardiography. Thus, because the patient had severe interstitial syndrome (lung ultrasound score 22), the etiology of their hypoxia was considered to be related to increased dead space and IPS.

## Discussion

The present study provides ‘proof of concept’ that combined lung ultrasound, DVT ultrasound, and limited TTE with saline microbubble contrast performed using handheld ultrasound may be able to define the etiology of hypoxia and shunt in patients with COVID-19 pneumonia.

Feasibility was demonstrated as interpretable images could be obtained in all participants and there was good inter-observer agreement. Importantly, no diagnostic errors in the reports were detected by an independent observer. Although one patient had grade 4 shunt, color Doppler failed to detect intracardiac shunt in any of our patients. Intravenous saline microbubble contrast was required to demonstrate this. The absence of any immediate, or delayed, complications following i.v. injection of saline microbubble contrast suggests that this procedure is safe in hypoxic, critically ill patients, but larger studies are required to confirm this.

Our observations confirm previous reports of lung ultrasound findings in COVID-19 pneumonia [12, 22, 27]. Our study also demonstrates that intrapulmonary, and interatrial shunt, and right ventricular dysfunction are present in select patients with COVID-19, and can be detected using a handheld ultrasound device.

Interatrial shunt is underdiagnosed in critically ill patients [11, 17, 18]. In a prospective study of 108 mechanically ventilated patients, using transesophageal echocardiography, patent foramen ovale (PFO) was detected in 27% overall [17]. However, when plateau pressure was over 26 cmH_2_O, the prevalence of PFO was significantly higher (46%) [17].

Patients with RTL extrapulmonary shunt respond poorly to positive end expiratory pressure (PEEP), are ventilated longer, receive more treatments for refractory hypoxia, and have longer admissions in ICU [5, 11, 18]. There are no data from randomized controlled clinical trials to guide management of patients with extrapulmonary shunt. Indeed, the standard approach to the management of refractory hypoxia, which aims to reduce IPS, can exacerbate RTL extrapulmonary shunt, and may even worsen hypoxia [5, 11, 17]. So, pathophysiology-directed treatment should aim to reduce total shunt (i.e., IPS and extrapulmonary shunt) [29].

However, the demonstration of interatrial shunt in a patient with RV failure highlights the complex and poorly understood relationship between interatrial shunt and cardiac function in critically ill patients. Left-to-right interatrial shunt can cause pulmonary hypertension and RTL interatrial shunt can cause hypoxia and platypnea-orthodeoxia [6, 30]. Thus, indications for closure of PFO and atrial septal defects are well established [30]. While successful closure of interatrial defect is oft reported [6, 11, 30, 31], it is important to be aware that acute cor pulmonale may develop after shunt closure [6, 11, 31], and that closure is not always required because RTL shunt may improve spontaneously as the functional trigger resolves [11]. Furthermore, atrial septostomy may be performed to decompress the left atrium to treat LV failure [32], and to decompress the right atrium in patients with chronic RV failure and pulmonary hypertension [33].

Thus, more data are required to define the most appropriate management strategies in hypoxic patients with interatrial shunt. As acute extrapulmonary shunt may be reversible, specific measures to identify, and where possible reduce or eliminate RTL extrapulmonary shunt may improve outcomes in select patients [5, 29].

Regardless, our observations suggest that combined lung ultrasound, DVT ultrasound, and limited TTE with saline microbubble contrast can define the etiology of shunt, and exclude DVT in hypoxic critically ill patients. When screening for the cause of hypoxia and shunt, these tools provide complementary data. The diagnostic information obtained can have an additive effect that provides new insights, improving decision-making.

Furthermore, our demonstration of the ease with which shunt can be detected could enhance the management of respiratory failure. Serial ultrasonography with handheld devices may allow monitoring of changes in lung pathology, shunt, and cardiac function after specific interventions, or with disease progression.

Further research is required to investigate the effects of specific causes of respiratory failure, and/or their treatment on total shunt (i.e., extrapulmonary shunt and IPS) [29, 34]. Our observations demonstrate that handheld ultrasound could facilitate such studies. Indeed, the COVID-19 pandemic offers a unique opportunity to advance the evidence base for critical care by studying patients with a unique etiology of respiratory failure [34]. Observations in COVID-19 may be relevant to other causes of respiratory disease [34].

Limitations of this study include the use of convenience rather than sequential sampling, which could cause inclusion bias. Our study only included select patients suspected to have severe shunt, and the detected shunts were relatively large. Further studies are required to determine whether handheld devices can detect small shunts in unselected critically ill patients.

Performance bias and overinterpretation by the investigators performing the ultrasound examinations was mitigated by a quality assurance check by an independent observer. This ensured that the images were interpretable, and that the correct diagnosis was made. No changes in diagnosis were made by the independent observer. The performance of imaging by experienced operators provides good internal validity. However, while this does limit external validity, intensivists with less imaging experience can ask a sufficiently experienced observer (e.g., cardiologist) to report their scans. Thus, this study provides a proof of concept.

## Conclusion

Routine screening of patients with combined lung ultrasound DVT ultrasound, and limited TTE with saline microbubble contrast using handheld ultrasound is feasible, and may be able to define the etiology of hypoxia and shunt in critically ill patients. The authors recommend further studies to determine whether using handheld ultrasound to characterize shunt, and direct management could improve the outcomes of critically ill patients. Observations in COVID-19 may be relevant to other causes of respiratory disease [5, 29, 34]. However, to increase generalizability, future research should consider subgroup analyses to explore outcomes based on RTL shunt, and RV function, as well as lung mechanics [5, 29, 34].

### Supplementary information

**Additional file 1: Video S1. **Intravenous microbubble contrast enhanced echocardiographic study suggesting intrapulmonary shunt. Labelled 2-D subcostal 4 chamber echocardiographic recording of the heart after i.v. injection of saline microbubble contrast demonstrating grade 3 shunt (> 25 bubbles). *RA* right atrium, *RV* right ventricle, *LA* left atrium, *LV* left ventricle. Bubbles appeared in the LA 5 cardiac cycles after opacification of the RA suggesting the presence of an intrapulmonary shunt.

**Additional file 2: Video S2. **Intravenous microbubble contrast enhanced echocardiographic study demonstrating interatrial shunt. Labelled 2-D subcostal 4 chamber transthoracic echocardiographic (TTE) recording of the heart after intravenous injection of saline microbubble contrast demonstrating grade 4 shunt (opacification of right atrium). Bubbles appeared in the left atrium (LA) within 3 cardiac cycles of opacification of the right atrium (RA) demonstrating the presence of an interatrial shunt. The RA and right ventricle (RV) are dilated and RV function is impaired. These findings are consistent with acute cor pulmonale. *LV* left ventricle.

## Data Availability

All authors affirm that this manuscript is an honest, accurate, and transparent account of the study being reported; that no important aspects of the study have been omitted; and that any discrepancies from the study as planned have been explained. The data that support the findings of this study are available from King Abdulaziz Medical City (KAMC), Ministry of National Guard – Health Affairs, Riyadh, Saudi Arabia. But restrictions apply to the availability of these data, which were used under license for the current study, and so are not publicly available. Data are however available from the authors upon reasonable request and with permission of KAMC.

## Abbreviations

COVID-19: Coronavirus Disease 2019
DVT: Deep vein thrombosis
TTE: Transthoracic echocardiography
ARDS: Acute respiratory distress syndrome
RTL: Right-to-left
HDU: High dependency unit
ICU: Intensive care units
IPS: Intrapulmonary
PaO2: Arterial partial pressure of oxygen
FiO2: Inspired oxygen fraction
PE: Pulmonary embolism
PFO: Patent foramen ovale
2-D: Two dimensional
LA: Left atrium
LV: Left ventricle
RA: Right atrium
RV: Right ventricle
