# Correction: Feasibility of using a handheld ultrasound device to detect and characterize shunt and deep vein thrombosis in patients with COVID-19: an observational study

**DOI:** 10.1186/s13089-023-00342-5

**Published:** 2023-11-23

**Authors:** Rajkumar Rajendram, Arif Hussain, Naveed Mahmood, Mubashar Kharal

**Affiliations:** 1 Department of Medicine, King Abdulaziz Medical City, King Abdullah International Medical Research Center, Ministry of National Guard - Health Affairs, Riyadh, Saudi Arabia; 2 College of Medicine, King Saud Bin Abdulaziz University for Health Sciences, Riyadh, Saudi Arabia; 3 Department of Cardiac Sciences, King Abdulaziz Medical City, King Abdullah International Medical Research Center, Ministry of National Guard - Health Affairs, Riyadh, Saudi Arabia

**Correction: Ultrasound J (2020) 12:49** **https://doi.org/10.1186/s13089-020-00197-0**

After publication of this article [1], the authors reported that in this article the affiliation details of affiliations 1 and 3 were incorrectly given as 'King Abdulaziz International Medical Research Center' but should have been 'King Abdullah International Medical Research Center'.

The original article [1] has been corrected.

## References

[1] Rajendram R, Hussain A, Mahmood N, Kharal M (2020). Feasibility of using a handheld ultrasound device to detect and characterize shunt and deep vein thrombosis in patients with COVID-19: an observational study. Ultrasound J.

